# Nanoporous Anodic Alumina: A Versatile Platform for Optical Biosensors

**DOI:** 10.3390/ma7064297

**Published:** 2014-05-30

**Authors:** Abel Santos, Tushar Kumeria, Dusan Losic

**Affiliations:** School of Chemical Engineering, The University of Adelaide, Engineering North Building, Adelaide 5005, Australia; E-Mails: tushar.kumeria@adelaide.edu.au (T.K.); dusan.losic@adelaide.edu.au (D.L.)

**Keywords:** nanoporous anodic alumina, electrochemical anodisation, structural engineering, chemical functionalisation, optical properties, optical biosensors

## Abstract

Nanoporous anodic alumina (NAA) has become one of the most promising nanomaterials in optical biosensing as a result of its unique physical and chemical properties. Many studies have demonstrated the outstanding capabilities of NAA for developing optical biosensors in combination with different optical techniques. These results reveal that NAA is a promising alternative to other widely explored nanoporous platforms, such as porous silicon. This review is aimed at reporting on the recent advances and current stage of development of NAA-based optical biosensing devices. The different optical detection techniques, principles and concepts are described in detail along with relevant examples of optical biosensing devices using NAA sensing platforms. Furthermore, we summarise the performance of these devices and provide a future perspective on this promising research field.

## 1. Introduction

Optical biosensors are able to accurately quantify small concentrations of analytes in milliseconds without sample contamination or alteration, since the sensing principle of these devices relies on the interaction between light and analyte molecules. Recently, the combination of different optical techniques (e.g., surface plasmon resonance, localized surface plasmon resonance, surface-enhanced Raman scattering, photoluminescence and reflectometric interference spectroscopy) with sensing platforms based on nanomaterials has received notorious attention. These nanostructures can confine, guide, transmit and enhance optical signals when they interact with light, as well as working as containers capable of accommodating and immobilising analyte molecules in a selective manner. This has enabled the design and development of ultra-sensitive optical biosensing systems for a broad range of analytes and applications (e.g., clinical analysis, environmental monitoring of pollutants, food contaminants, *etc.*) [1]. Amid the different nanomaterials used to develop optical sensing platforms, nanoporous anodic alumina (NAA) has gained increasing interest during the last few years as a result of its interesting physical and chemical properties. NAA is an optically active material produced by the electrochemical anodisation of aluminium, the pores of which act as nanocontainers and selective filters for analyte molecules by size-exclusion and surface chemistry selectivity. Pore geometry in NAA can be structurally engineered by different electrochemical approaches. This makes it possible to engineer and design the effective medium of NAA in order to produce a broad range of optically active structures (e.g., omnidirectional mirrors, microcavities, distributed Bragg reflectors, waveguides, rugate filters, *etc.*) [2]. Furthermore, surface chemistry in NAA can be modified through well-established protocols in order to selectively target a broad range of analyte molecules [3].

In this scenario, this review is aimed at compiling and summarising the most relevant advances and developments of optical biosensors based on NAA. First, we will introduce a brief description of the fabrication process of NAA and the different electrochemical approaches used to develop optical platforms based on NAA. Second, we will describe the most relevant and recent developments of optical biosensing systems based on NAA, providing a detailed description about their sensing principles, performances and practical applications. Finally, we will provide a prospective outlook on this field.

## 2. Fabrication and Structural Engineering of NAA

### 2.1. Fabrication of NAA

**Figure 1:**
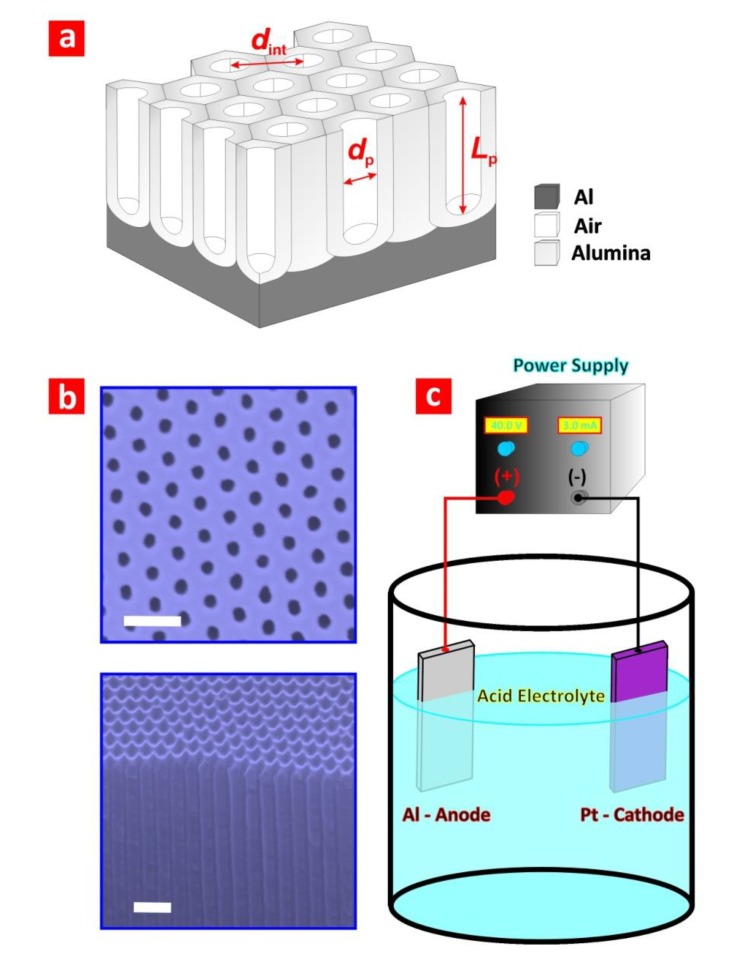
(**a**) Illustrative scheme describing the most representative geometric features of nanoporous anodic alumina (NAA) (*i.e.*, pore diameter, pore length and interpore distance); (**b**) top and cross-section scanning electron microscopy (SEM) images of NAA (scale bars = 400 and 250 nm, respectively); (**c**) illustration describing a basic electrochemical anodisation cell used to produce NAA.

NAA is produced by electrochemical anodisation of aluminium in different acid electrolytes (e.g., oxalic, sulphuric, phosphoric, *etc.*). Since the first decades of the 20th century, this electrochemical process has been intensively used for a broad range of industrial applications, including surface finishing, automobile engineering, machinery, corrosion protection, and so on [4]. The use of electronic microscopes in the 1950s revealed the nanoporous structure of NAA and made it possible to establish the effect of the different fabrication parameters on the physical and chemical properties of NAA [5,6,7,8,9,10]. In that regard, the most outstanding milestone in the use of NAA in nanotechnology was reported by Masuda and Fukuda by introducing the two-step anodisation process [11,12,13,14]. In this electrochemical approach, the resulting nanoporous oxide layer is chemically removed after the first anodisation step in order to pattern the surface of the aluminium substrate. This enables the propagation and self-organised growth of cylindrical nanopores from top to bottom during the course of the second anodisation step. Highly ordered NAA structures can be produced by this simple, cost-effective and brilliant strategy, which does not require sophisticated or expensive laboratory facilities as compared to traditional lithographic methods. Self-ordered NAA consists of a nanoporous matrix based on alumina (aluminium oxide (Al_2_O_3_)) featuring close-packed arrays of hexagonally arranged cells containing a cylindrical nanopore at the centre, which grows perpendicularly to the underlying aluminium substrate (Figure 1a,b). The electrochemical anodisation of aluminium is typically carried out in acid electrolytes consisting of aqueous solutions of sulphuric (H_2_SO_4_), oxalic (H_2_C_2_O_4_) or phosphoric acids (H_3_PO_4_), in which both electrodes, the anode (*i.e.*, aluminium foil (Al)) and cathode (e.g., platinum wire (Pt)), are immersed (Figure 1c). When a given anodisation voltage is applied between the anode and cathode, pores nucleate and start to grow perpendicularly to the Al surface. The pore growth mechanism in steady state is the result of competing oxidation (*i.e.*, the formation of oxide) and dissolution (*i.e.*, the dissolution of oxide) throughout the anodisation process. First, aluminium oxide grows at the aluminium-alumina interface, due to the countermigration of ionic species (*i.e.*, Al^3+^ and O^2−^) through the oxide barrier layer. Second, Al_2_O_3_ is dissolved at the alumina-electrolyte interface. This electrochemical process can be described by the following reduction-oxidation (*i.e.*, redox) equations:

(i) Formation of alumina (aluminium-alumina interface (anode))
2Al(s) + 3H_2_O(1) ↔ Al_2_O_3_(s) + 6H^+^(aq) + 6e^−^ (1)
(ii) Dissolution of alumina (alumina-electrolyte interface (anode))
Al_2_O_3_(s) + 6H^+^(aq) → 2Al^3+^(aq) + 3H_2_O(1) (2)
(iii) Diffusion of aluminium cations (within oxide barrier layer (anode))
2Al(s) → 2Al^3+^(aq) + 6e^−^ (3)
(iv) Hydrogen evolution (electrolyte-cathode interface (cathode))
6H^+^(aq) + 6e^−^ → 3H_2_(g) (4)

It is worthwhile noting that other side reactions (e.g., oxygen evolution at the anode) evolve throughout the anodisation process. For this reason, the experimental anodic current efficiency is always lower than 100%.

Depending on the anodisation conditions, aluminium can be anodised under mild (MA) or hard anodisation (HA) regimes [4,11,12,13,14]. While the former is carried out at moderate voltages and temperatures, the latter is performed at high voltages and low temperatures. Notice that the different anodisation conditions for each of these regimes will be established by the acid electrolyte. Table 1 summarises the most representative MA and HA anodisation conditions, as well as the geometric features of the resulting NAA. Another difference between NAA produced under MA and HA conditions is the pore growth rate, which is constant and slow in the MA regime (*i.e.*, from 3 to 8 µm·h^−1^) and exponentially decreasing and fast in the HA regime (*i.e.*, from 50 to 70 µm·h^−1^). Pore geometry in NAA can be defined by such structural parameters as pore diameter (*d*_p_), pore length (*L*_p_) and interpore distance (*d*_int_) (Figure 1a). These parameters can be accurately designed by the anodisation conditions in the range of 10–400 nm for *d*_p_, from several nanometres to hundreds of micrometres for *L*_p_ and 50–600 nm for *d*_int_. The effect of the different anodisation parameters on the physical and chemical properties of NAA has been intensively studied during the last few decades [15]. In that regard, the anodisation voltage/current and the electrolyte type are recognised as the most critical parameters to control the pore geometry of NAA.

**Table 1:** Summary of the most representative fabrication conditions for NAA produced in the mild anodisation (MA) and or hard anodisation (HA) regimes and the geometric characteristics of the resulting nanostructures.

Acid electrolyte	Anodisation regime	*V* (V)	*T* (°C)	*d*_p_ (nm)	*d*_int_ (nm)	Growth rate (µm·h^−1^)	References
H_2_SO_4_ 0.3 M	MA	25	5–8	25	63	7.5	[12]
H_2_SO_4_ 0.3 M	HA	40	0–1	30	78	85	[18]
H_2_C_2_O_4_ 0.3 M	MA	40	5–8	30	100	3.5	[11]
H_2_C_2_O_4_ 0.3 M	HA	140	0–1	50	280	50	[4]
H_3_PO_4_ 0.1 M	MA	195	0–1	160	500	2	[13]

**Figure 2:**
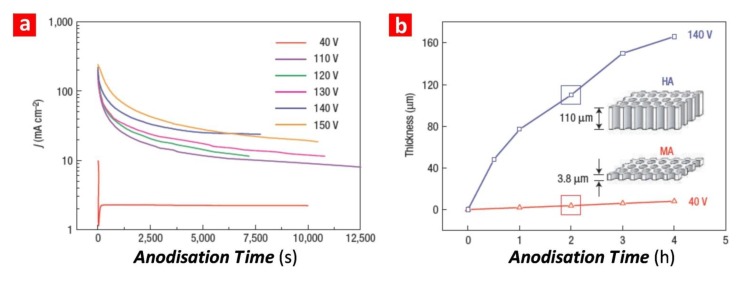
(**a**) Comparison of the current density profiles under MA and HA conditions for NAA produced in oxalic acid (adapted from [4]); (**b**) the growth rate under MA and HA conditions for NAA produced in oxalic acid (adapted from [4]).

In terms of the growth mechanism, the anodization process of aluminium in the MA regime is rate-limited by the ionic transport of ionic species (*i.e.*, Al^3+^ and O^2−^) across the aluminium-alumina interface, the alumina barrier layer and the alumina-electrolyte interface [16,17]. However, in the HA regime, this process is controlled by the mass transport of oxygen-containing anionic species (*i.e.*, HO^−^ and O^2−^) from the bulk electrolyte to the reaction interface (*i.e.*, the alumina-aluminium interface) [4]. This phenomenon is translated into the MA and HA profiles (Figure 2a). For this reason, while the pore growth rate in MA is nearly constant, it becomes exponentially decreasing in HA (*i.e.*, the longer the pore length, the slower the mass transport of oxygen-containing anionic species from the bulk electrolyte to the reaction interface) (Figure 2b).

**Figure 3:**
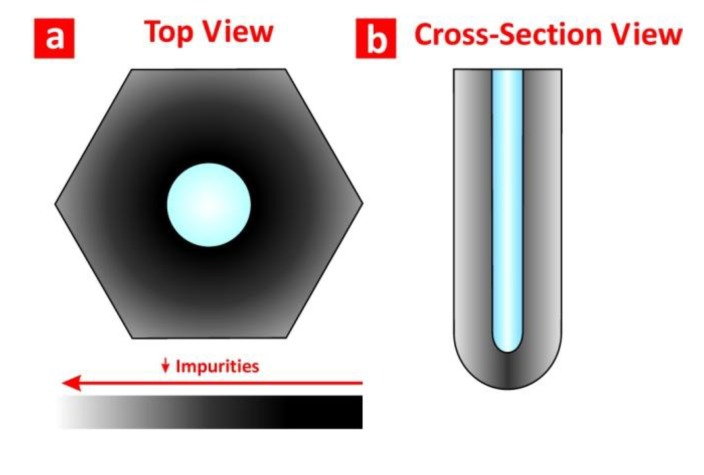
(**a**) Top view; and (**b**) cross-section illustrations showing the distribution of impurities in an NAA pore cell.

As for its chemical structure, NAA presents an onion-like structure distributed in a layered fashion (Figure 3). Previous studies established that this chemical structure is composed of an outer layer close to the central pore and an inner one far from it [14]. The former is composed of aluminium oxide (Al_2_O_3_) contaminated with impurities incorporated into the NAA structure from the acid electrolyte during the anodisation process. The latter, however, is basically composed of pure Al_2_O_3_. Nevertheless, it is worth stressing that, as other studies have pointed out, the real chemical structure of NAA is composed of more than two chemical layers. For instance, Yamamoto *et al*. [19] analysed the chemical structure of NAA by studying its photoluminescence spectrum after discontinuous chemical etching steps of its structure. This study revealed that the real chemical structure of NAA is composed of three chemical layers. More recently, we monitored in real-time the chemical dissolution of NAA by reflectometric interference spectroscopy [20]. We found out that the real chemical structure of NAA is composed of four chemical layers, with a decreasing concentration of impurities from the outer side of the pore walls (*i.e.*, close to the central pore) to the inner one (*i.e.*, far from the central pore).

The optical properties of NAA are directly related with its pore nanoporous structure (*i.e.*, pore diameter, pore length and interpore distance) and its chemical composition (*i.e.*, type, content and distribution of impurities). Therefore, the fabrication conditions and the structural design of the NAA structure become crucial factors to produce optical sensing platforms based on NAA with enhanced capabilities for biosensing applications.

### 2.2. Structural and Optical Engineering of NAA

**Figure 4:**
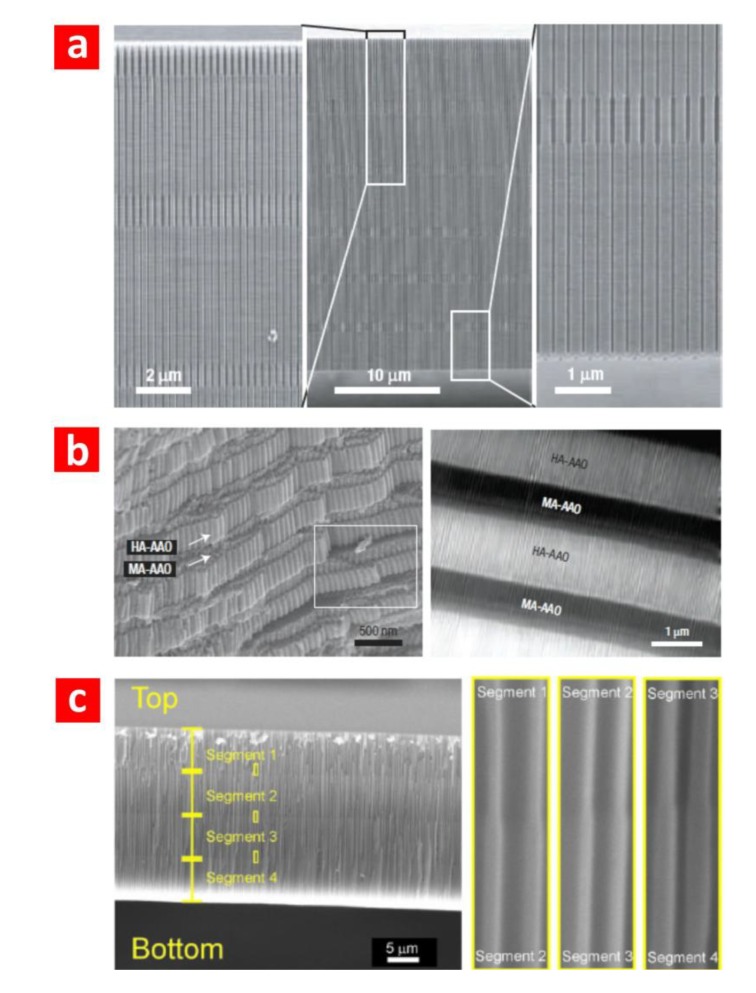
(**a**) Cross-section SEM images of pore diameter modulations in NAA introduced by Lee *et al.* by switching MA and HA regimes (adapted from [4]); (**b**) cross-section SEM and optical images of multilayered NAA produced by Lee *et al.* by pulse anodisation (adapted from [29]); (**c**) cross-section SEM images of funnel-like NAA produced by Santos *et al.* by combining multiple anodisation and pore widening steps in a sequential fashion (adapted from [38]). AAO, anodic aluminium oxide.

The nanoporous structure of NAA is a key parameter to design and produce a broad range of optical nanostructures with exquisitely designed optical properties. Light can be confined, guided, transmitted, emitted and reflected when it interacts with nanostructures based on NAA. So far, different electrochemical approaches have made it possible to engineer the structure of NAA to produce a new generation of nanostructures with potential applicability in optical biosensing. Some examples of NAA pore architectures are modulated [18,21,22], hierarchical [23,24], serrated [25,26], three-dimensional [27,28], multilayered [29,30,31,32,33] and funnel-like [34,35,36,37,38,39]. Table 2 summarises the most representative examples of NAA structures with potential applicability for developing optical sensing devices. Figure 4 shows images of these NAA structures generated by different electrochemical approaches.

**Table 2:** Summary of the most representative pore geometries in NAA produced by different electrochemical approaches.

Type of NAA	Electrochemical approach	References
Modulated	modulated anodisation profile	[18,21,22]
Hierarchical	asymmetric two-step anodisation	[23,24]
Serrated	anodisation at low voltage and high temperature	[25,26]
Three-dimensional	modulated anodisation profile and final wet chemical etching	[27,28]
Multilayered	stepwise or pulse anodisation	[29,30,31,32,33]
Funnel-like	combination of multiple anodisation and pore widening steps	[34,35,36,37,38,39]

Electrochemical approaches aimed at producing pore diameter modulations in NAA have been intensively researched due to the interesting optical properties of the resulting NAA structures. The most widespread method used to generate pore diameter modulations in NAA is to switch the anodisation regime from MA and HA in an alternating fashion (Figure 4a). Pore diameter modulations in NAA were pioneered by Lee *et al.* on the basis of the different levels of porosity between MA and HA in NAA produced in oxalic acid (*i.e.*, 10% for the former and 3% for the latter) [4]. In this study, the surface of the electropolished aluminium substrates was patterned by a master stamp featuring hexagonally arranged arrays of nanopyramids, the period of which was 275 nm. The first anodisation step was started under MA conditions in an aqueous solution of H_3_PO_4_ 0.4 M at 110 V and 10 °C for 15 min. Next, the acid electrolyte was replaced by an aqueous solution of H_2_C_2_O_4_ 0.015 M, and the anodisation voltage was set to 137 V. The electrolyte temperature was kept at 0.5 °C during this anodisation step to prevent NAA from burning. The time length of this step was 2 min. Highly uniform periodic modulations of the pore diameter in NAA were obtained by repeating this procedure. Notice that, in this process, the interpore distance in both layers (*i.e.*, MA and HA layers) remains constant due to the anodisation voltages being set to yield the same interpore distance in the MA and HA conditions. This prevented NAA from pore branching. The pore diameter of each segment was established by the acid electrolyte and the anodisation voltage, while the length of each segment was controlled by the anodisation time, making it possible to modulate the pore diameter in depth. This work was the origin of a flood of studies about different electrochemical approaches to generate pore diameter modulations in NAA [18,21,22].

Multilayered NAA structures consist of stacks of layers of NAA featuring different levels of porosity [29,30,31,32,33] (Figure 4b). This type of NAA can be fabricated by alternating the voltage during the anodisation process. Different anodisation profiles can be applied (e.g., stepwise, sinusoidal, pseudosinusoidal, saw-like, *etc.*) in order to engineer the effective medium of NAA in depth [29,30,31,32,33]. The resulting NAA nanostructures can be used to develop such active optical nanostructures as distributed Bragg reflectors, microcavities, rugate filters, omnidirectional mirrors and waveguides. In other words, the interaction between the incident light and the NAA nanostructure can be designed for subsequent applications, such as biosensors. Lee *et al.* [29] reported on an electrochemical approach for the continuous structural engineering of NAA through pulse anodisation under potentiostatic conditions in sulphuric acid. Periodic pulses consisting of low and high potential pulses between MA and HA conditions were applied to design and tailor the nanoporous structure and chemical composition of NAA along the pore axes. Taking advantage of this approach, Sulka *et al.* [30] demonstrated a distributed Bragg reflector (DBR) based on NAA. The resulting NAA layers of that nanostructure had different porosities and, thereby, different refractive indices, enabling the optical engineering of sensing platforms. Furthermore, they infiltrated selected areas on the NAA surface with polymers by a lithographic approach using transmission electron microscopy grids. The resulting DBR mirrors reflected light in two different ranges of wavelength. Zheng *et al.* [31] prepared DBRs based on NAA by anodising aluminium foils by a pseudosinusoidal profile under potentiostatic conditions. They demonstrated that the transmission peak of these DBRs can be effectively modulated to cover almost the whole visible spectrum by adjusting the anodisation temperature. Santos *et al.* [32] studied the effect of the anodisation voltage ramp and the hard anodisation voltage on the pore rearrangement during the MA to HA regime transition in bilayered NAA structures. Furthermore, the resulting nanostructures could be used to develop photonic crystals or Fabry–Pérot interferometers. More recently, Rahman *et al.* [33] used an innovative cyclic anodisation approach under potentiostatic conditions to produce DBRs based on NAA. They analysed the effect of pore widening on the photonic stop-band of the resulting nanostructures. This study demonstrated that the photonic stop-band of these optical nanostructures can be modulated by the pore widening time.

Another structure based on NAA with interesting optical properties is the so-called funnel-like NAA. This nanostructure is produced by combining multiple anodisation and pore widening steps in a sequential fashion [34,35,36,37,38,39]. In this procedure, the second anodisation step is substituted by multiple and sequential anodisation and pore widening steps. Funnel-like NAA can be described as a stack of layers of cylindrical nanopores featuring a decreasing pore diameter from top to bottom (Figure 4c). While the length of each layer is controlled by the anodisation time or the total charge (*i.e.*, an integrated current passed through the system), the pore diameter in that segment is engineered by the pore widening time. This enables the structural engineering of the nanoporous structure of NAA in depth and the replication of nanostructures based on different materials with optical properties. For example, He *et al.* [34] developed a suspension array for multiplexed immunoassays using shape-coded silica nanotubes as coding materials. These nanotubes were replicated from a funnel-like NAA template. Nagaura *et al.* [35] fabricated funnel-like NAA with a low aspect ratio and engineered its structure by changing the number of repeating cycles of anodisation and pore widening steps. In an extension of this work, the same authors created nickel replicas from these NAA templates and observed that the surface morphology of the nanoconical film exhibited the same periodic structure of the NAA template [36]. Using a similar approach, Yanagishita *et al.* [37] developed antireflection nanostructures based on polymer by photoimprinting funnel-like NAA moulds featuring different pore shapes. The reflectance of the obtained antireflection polymer nanostructures was assessed through the measurement of transmittance. Among the different nanostructures evaluated, they found that those featuring a gradually changing slope exhibited the lowest reflectance. Santos *et al.* [38] produced a high aspect ratio funnel-like NAA featuring two, three and four segments. The length of each segment was controlled by the total current charge passed through the system, making it possible to design the resulting NAA structure with a high degree of accuracy. More recently, Li *et al.* [39] reported on an electrochemical approach to tailor the shape of funnel-like NAA structures. They demonstrated a precise control of the resulting nanostructure (*i.e.*, linear cones, whorl-embedded cones, funnels, pencils, parabolas and trumpets) by the fabrication parameters (*i.e.*, anodisation time, etching time and cyclic times. Typically, funnel-like NAA features well-defined cylindrical nanopores with decreasing pore diameter from top to bottom. However, we have recently reported on a unique approach to produce inverted funnel-like NAA [20]. This nanostructure results from combining multiple anodisation steps and annealing treatments with a final pore widening step. In contrast to funnel-like NAA, inverted funnel-like NAA features an increasing pore diameter from top to bottom.

## 3. Optical Biosensors Based on NAA

As the aforementioned studies have demonstrated, NAA has become an attractive material to develop optically active nanostructures due to its geometry and chemical composition. Other materials, such as porous silicon, have been intensively used to develop a broad range of optical devices, including biosensors, due to its outstanding optical and electronic properties [40,41]. However, porous silicon has to be passivated through a laborious process in order to achieve stable optical signals, since its structure is degraded under biological conditions. In contrast, NAA is chemically and mechanically stable under these conditions, providing stable optical signals throughout without further passivation [1]. Other capabilities of NAA are that its nanoporous structure can be engineered through different electrochemical approaches and its surface chemistry modified through well-established protocols [42,43], which are fundamental aspects for developing highly sensitive and selective optical biosensors. Within this context, a new generation of optical sensing devices based on NAA have recently emerged, and their sensing performance has successfully been explored for many analytical applications. The obtained results have demonstrated that NAA is a promising alternative to biosensing platforms based on porous silicon. The most representative advances in development and applications of optical NAA biosensors will be described throughout the following sections (Table 3).

**Table 3:** Summary of optical biosensors based on NAA platforms: detection principle, application and sensing performance. SPR, surface plasmon resonance; SERS, surface-enhanced Raman scattering; PL, photoluminescence; RIfS, reflectometric interference spectroscopy.

Sensing Platform	Analyte	Detection Limit	References
SPR-NAA	avidin	10 µg·mL^−1^	[44]
	anti-5-fluorouracil	100 mg·mL^−1^	[44]
	melittin	100 ng·mL^−1^	[44]
	Ru[BPhen_3_]^2+^	2 µM	[45]
	Fe[Phen_3_]^2+^	1 µM	[45]
	BSA	60 nM	[46]
	invertase	10 nM	[36]
SERS-NAA	p-aminothiophenol	0.5 M	[47]
	4-mercaptopyridine	1 × 10^−6^ M	[48]
	3-mercaptobenzoic	3 mM	[49]
	benzenethiol	500 ppb	[50]
	N-methyl-4-nitroaniline	3 ppb	[50]
PL-NAA	morin	5 × 10^−6^ M	[51]
	trypsin	40 µg·mL^−1^/0.1 mg·mL^−1^	[51,52]
	DNA	100 mM	[53]
	oxazine 170	6.5 × 10^−3^ M	[54]
	glucose	0.1 M	[54]
	glucose	10 mM	[55]
	cysteine	5 mM	[55]
RIfS-NAA	H_2_S	0.5 v%	[56]
	DNA	2 nmol·cm^−2^	[57]
	circulating tumour cells	1000 cells·mL^−1^	[58]
	immunoglobulin	0.1 mg·mL^−1^	[59]
	glucose	100 mM	[55]
	cysteine	5 mM	[55]
	gold(III)	0.1 µM	[60]
	BSA	1 mg·mL^−1^	[61]
	glucose	0.01 M	[2]
	BSA	15 µM	[58]
	human IgG	600 nM	[62]

### 3.1. Surface Plasmon Resonance (SPR)

**Figure 5:**
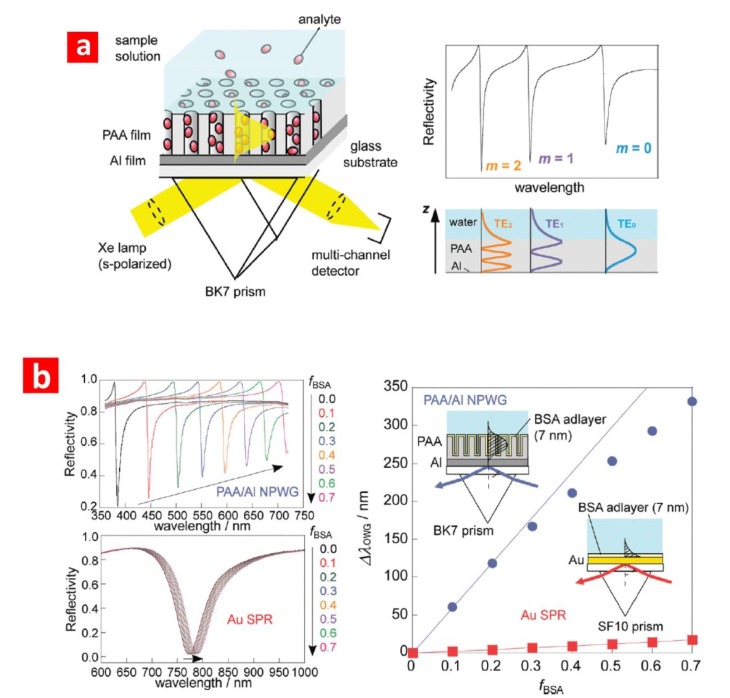
(**a**) Schematic illustration of the SPR-NAA sensor developed by Hotta *et al.* and the typical reflection spectrum of this system with the electric field distributions of the TE waveguide modes with different mode orders (*m*) (adapted from [45]); (**b**) changes in reflection spectra simulated by the Fresnel calculations depending on the surface coverage of BSA molecules (*f*_BSA_) (adapted from [45]). TE, transverse electric; PAA, porous anodic alumina; NPWG, nanoporous optical waveguide.

SPR is an optical technique based on the generation of surface plasmons by an evanescent electromagnetic wave, which takes place when light is shined on the surface of a prism coated with metal. Typically, this system is implemented in a Kretschmann configuration, where an NAA layer is grown by anodising a thin aluminium film grown on the prism [63]. The plasmonic properties of this system are strongly dependent on the effective refractive index of the adjacent medium (*i.e.*, NAA film) within distances of several hundreds of nanometres. Therefore, any change in the effective medium inside the NAA matrix can be quantified. As a result of its outstanding capabilities, SPR is one of the most widespread analytical methods used to detect biological events (e.g., adsorption/desorption of molecules, digestion of proteins, association/dissociation and specific ligand-ligand interactions). This technique is capable of monitoring any dynamic change in the effective medium of NAA in real time and *in situ*. Furthermore, a suitable design of the nanoporous structure of NAA makes it possible to confine and guide light and to minimise scattering losses, which are key parameters to develop optical systems with enhanced sensitivity [45]. Figure 5 shows a typical SPR system based on NAA used to develop optical biosensors. Lau *et al.* [46] combined SPR with NAA to develop an optical biosensing platform capable of characterising the adsorption and desorption of bovine serum albumin (BSA) at different values of pH. A similar study was carried out by Koutsioubas *et al.* [64], who characterised the adsorption and desorption of BSA onto a SPR-NAA system at different values of pH. Dhathathreyan demonstrated that this system is able to quantify real-time biological events, such as enzymatic reactions [65]. In this study, enzyme invertase was immobilised onto the nanoporous network of NAA, and its activity at different pHs and substrate concentrations was established when digesting sucrose to produce glucose and fructose. SPR results revealed biphasic kinetics for both the adsorption of the enzyme and its interaction with the substrate. Lau *et al.* [66] used a SPR-NAA system to study the grafting of poly(g-benzyl-L-glutamate) (PBLG) within the nanopores of NAA. They compared the results obtained in NAA with a planar silicon dioxide surface. These revealed that the conformation of the PBLG nanostructure within NAA was associated with the confining of polymer chains within the nanopores of NAA. Hotta *et al.* [45] engineered the structure of NAA to demonstrate that this parameter can significantly improve the sensitivity of SPR-NAA systems. In this study, the sensing performance of SPR-NAA with optimised pore geometry was assessed by monitoring the adsorption of BSA molecules onto the nanoporous network of NAA. They observed a larger red shift (>300 nm) of this optical signal as compared to other SPR-NAA systems, which was associated with the optimised nanoporous structure of NAA. Another type of widely used SPR-NAA system is the localised surface plasmon resonance (LSPR) system, where highly ordered arrays of metallic nanoparticles, typically of gold or silver, are deposited or assembled on the top or bottom surfaces of NAA. As a result of the hexagonally arranged structure of NAA at the nanoscale, LSPR-NAA systems can provide outstanding sensing capabilities. For example, Hiep *et al.* [44] deposited gold nanoparticles on the top surface of NAA and developed an LSPR-NAA system to detect specific interactions of such biomolecules as biotin/avidin and 5-fluorouracil/anti-5-fluorouracil. This systems showed a considerable enhancement of the sensing capabilities due to the multiple interactions of incident light and gold nanoparticles, which occurred when a suitable matching condition between LSPR and interference spectra were established.

### 3.2. Surface-Enhanced Raman Scattering (SERS)

Surface-enhanced Raman scattering (SERS) is a surface-sensitive optical technique that enhances Raman scattering by molecules adsorbed on rough metal surfaces or nanostructures. The most likely mechanism that explains the enhancement effect of SERS is associated with the excitation of localised surface plasmons. Nevertheless, the real mechanism of SERS is still a matter of debate. The SERS effect is translated into an enhancement of the order of 10^6^ of the Raman signal from molecules adsorbed onto the surface of a metal substrate with nanometric roughness, known as “hot spots” or “hot junctions”. SERS is an ultra-sensitive technique able to detect, identify and quantify trace amounts of analyte molecules. NAA has been demonstrated to be an excellent platform to develop SERS substrates, due to its organised nanostructure and cost-competitive and scalable fabrication process. Typically, SERS-NAA platforms are fabricated by sputtering or evaporating metals, basically gold and silver, on the top or bottom surface of NAA substrates, although other options, such as embedded nanowires, decorative nanoparticles and metallic membranes, have been explored, too. As a result of its versatility in terms of geometry, SERS-NAA platforms can be broadly engineered by the anodisation parameters and the metal deposition conditions to provide optimised SERS signals for specific sensing applications. SERS-NAA platforms featuring gold or silver nanoparticles on their surface have been demonstrated to provide strong Raman signal enhancements of up to the order of 10^6^.

**Figure 6:**
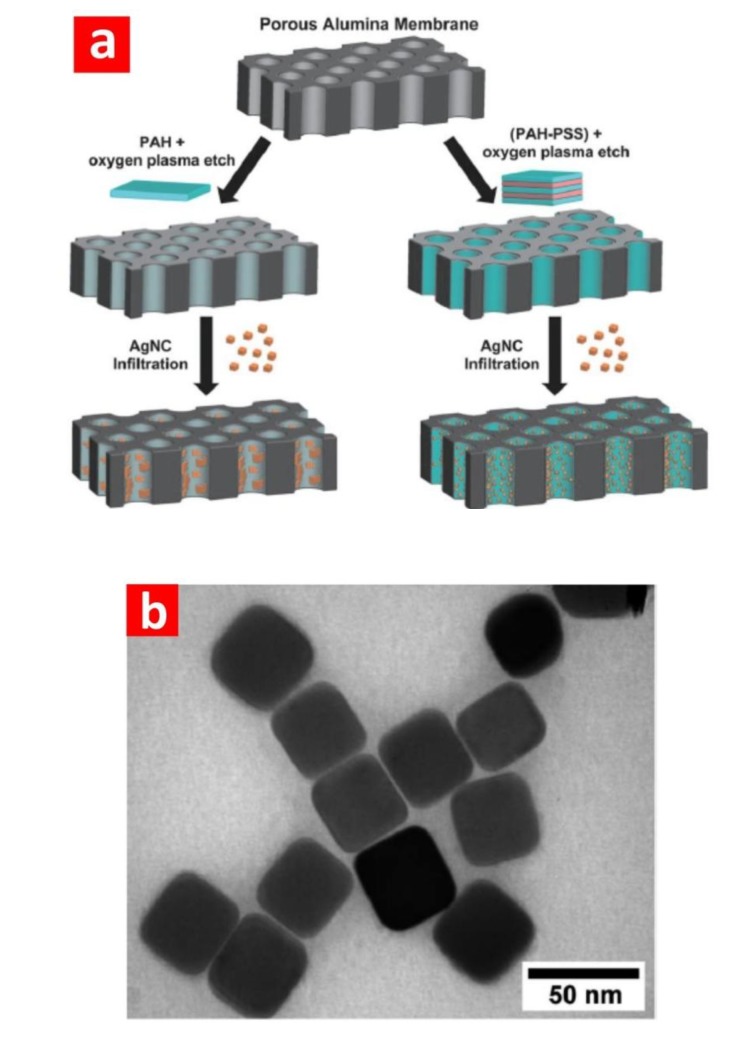
(**a**) Schematic of the silver nanocube infiltration method showing the NAA membrane coated with a positively charged polyelectrolyte (PAH, PEI or PAH–PSS bilayers) that is subsequently decorated with silver nanocubes by infiltration (adapted from [50]); (**b**) transmission electron microscopy image of silver nanocubes (adapted from [50]). PAH, poly(allylamine hydrochloride); PEI, polyethylenimine; PSS, poly(sodium 4-styrenesulfonate).

For instance, Ko *et al.* [67] decorated the nanopores of NAA with gold nanoparticles and demonstrated the sensing capability of this sensing platform by detecting trace amounts of 2,4-dinitrotoluene, which is a nitroaromatic compound of TNT-based plastic explosive non-detectable by the conventional Raman technique. Lu *et al.* [47] used silver nanoparticles to decorate the nanopores of NAA by incubation and subsequent reduction of Ag(NH_3_)^2+^. The size and density of silver nanoparticles was tuned to optimise SERS signals. The sensing performance of this SERS-NAA platform was assessed by detecting *p*-aminothiophenol. A point-by-point SERS mapping of this analyte on the NAA platform was acquired to characterise the homogeneity of the adsorbed molecules. Ji *et al.* [48] used a similar strategy to detect and characterise 4-mercaptopyridine on SERS-NAA platforms decorated with electrodeposited silver nanoparticles. Different deposition times were investigated in order to assess the homogeneity and stability of these SERS platforms. Notice that the use of NAA as a SERS substrate is not limited to decoration with gold or silver nanoparticles. Other studies have demonstrated that metallic nanowires and nanotubes embedded in NAA templates can provide outstanding SERS signals, as well. For example, Lee *et al.* [68] grew silver nanowires inside NAA platforms by electrodeposition and demonstrated their potential to develop SERS platforms. In this study, the effect of the interwire gap distance (*i.e.*, the distance between adjacent nanowires in the NAA matrix) was assessed when detecting 4-aminobenzenethiol. They found that the SERS intensity signal can be increased over 200-fold when the interwire gap distance is reduced from 35 to 10 nm. Another type of SERS-NAA platform was developed by Valleman *et al.* [49], who carried out the electroless deposition of gold inside the NAA template. The resulting gold/NAA composite membrane was employed in order to confirm and characterise the formation of monolayers of 3-mercaptobenzoic acid. They observed that the slight curvature of gold within the nanopores increased the SERS effect along the pores of the SERS-NAA platform, with increasing Raman peak intensities from the middle of the nanopores towards their bottom. More recently, Kodiyath *et al.* [50] decorated NAA templates with silver nanocubes by an approach based on polyelectrolytes (Figure 6). The resulting SERS-NAA platforms were used to carry out the efficient, robust, tuneable and reusable sensing of trace levels of organic vapour. They demonstrated that the detection limit and the signal saturation can be tuned by modifying the number of polyelectrolyte bilayers on the pore walls of the NAA templates. The sensing performance of these SERS-NAA platforms was assessed by detection benzenethiol and N-methyl-4-nitroaniline. They observed that this was highly dependent on the distribution of silver nanocubes. A comparison between silver nanocubes and nanospheres was carried out in this study, demonstrating that the former provide better sensing performance for the aforementioned analytes.

### 3.3. Photoluminescence (PL)

Although the photoluminescence (PL) properties of NAA were reported several decades ago and extensive studies have been carried out since then, their origin is still in doubt [69,70,71]. It is generally accepted, however, that PL in NAA is dependent on two types of PL centres: namely; (i) F centres related to the amount of impurities incorporated into the NAA structure from the acid electrolyte during the anodisation process [72]; and (ii) F^+^ centres associated with ionised oxygen vacancies in the amorphous structure of NAA [70,71]. The PL properties of NAA rely upon such fabrication parameters as acid electrolyte, anodisation voltage and regime and thermal treatments [69,73,74]. Pioneering studies on PL-NAA platforms used the nanopores of bare NAA templates to accommodate and detect analyte molecules by comparing the PL spectrum of NAA before and after analyte infiltration. These studies demonstrated that physically adsorbed molecules can be detected through shifts in the PL spectrum of NAA. For example, Jia *et al.* [51] used PL-NAA sensing platforms to detect non-fluorescent morin and morin-trypsin molecules through changes in the PL spectrum of NAA. From these measurements, they inferred that chemical interactions between the NAA structure and morin molecules take place during this process, which were associated with the observed shifts in the PL band of NAA after infiltration.

**Figure 7:**
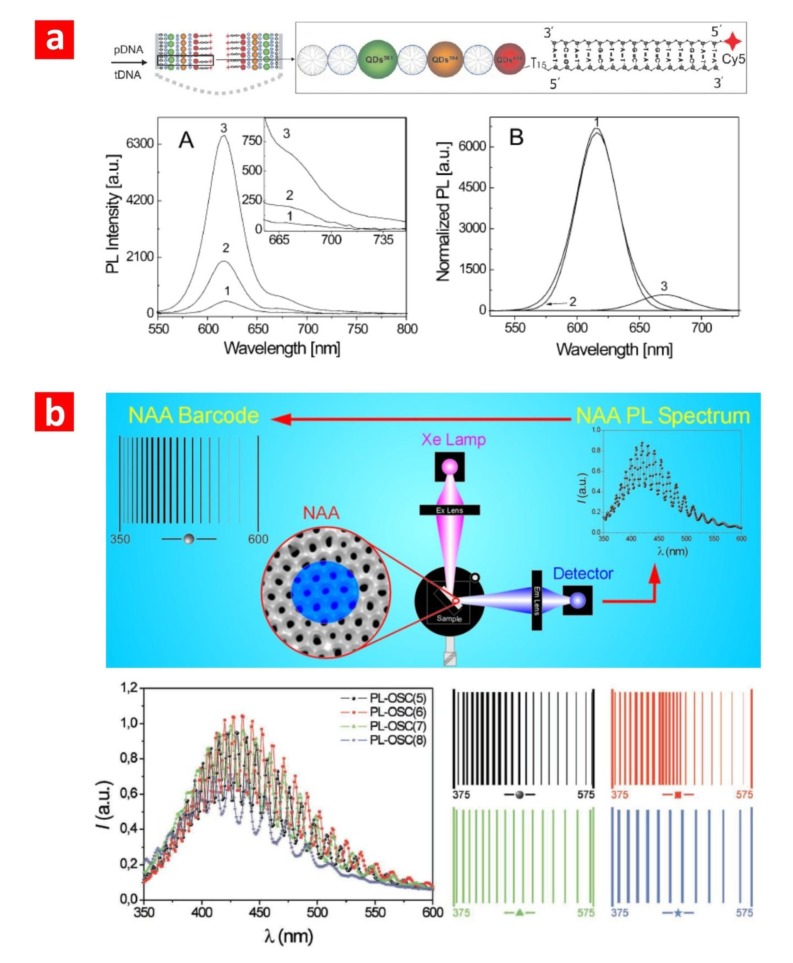
(**a**) PL-NAA biosensing platform developed by Feng *et al.* to monitor DNA hybridisation (A, Normalized PL spectra of this biosensing platform after DNA hybridisation; B, Deconvoluted PL spectrum shown in A) (adapted from [53]); (**b**) optical barcode system based on PL-NAA biosensing platforms developed by for Santos *et al.* (adapted from [52,54]).

Feng *et al.* [53] used a more sophisticated approach to monitor the hybridisation of DNA molecules through PL changes in NAA platforms modified with quantum dots (Figure 7a). In their study, the surface chemistry of NAA was functionalised with mercaptoundecanoic acid followed by layer-by-layer deposition of positively and negatively charged dendrimers and ZnCdSe quantum dots. This PL-NAA system showed enhanced sensitivity afforded by the photoluminescent quantum dots, which provided the ability to detect small changes of the PL signal associated with DNA hybridisation. The sensitivity was found to be tuneable by modifying the assemblies of quantum dots. Santos *et al.* [54] developed a sophisticated optical barcode system based on the PL of NAA in the UV-visible region. The PL spectrum of NAA presents an abundance of narrow, well-resolved oscillations generated by the Fabry–Pérot effect, which amplifies and enhances the PL of NAA at wavelengths corresponding to the optical modes of the cavity formed by the system, air-NAA-aluminium. These oscillations constitute the base of this optical biosensing system. It is worth mentioning that the number, intensity and position of these oscillations can be tuned by modifying the pore length and diameter of NAA (*i.e.*, by changing the effective medium). These PL-NAA platforms have been tested with success for detecting biological substances, such as organic dyes, enzymes and glucose (Figure 7b) [52,54].

### 3.4. Reflectometric Interference Spectroscopy (RIfS)

Reflectometric interference spectroscopy (RIfS) is a highly sensitive optical sensing technique based on the interaction of white light with thin films [75]. In the past decade, Sailor and co-workers combined porous silicon platforms with RIfS to develop highly sensitive optical biosensors [76,77,78]. These have emerged as more efficient, attractive substitutes to traditional planar thin films used in earlier studies [79,80]. Following this success, NAA has recently been considered as an alternative substrate to porous silicon, offering several advantages, such as better chemical and mechanical stability and a more controllable and defined nanoporous structure [59]. NAA presents an RIfS spectrum with well-resolved peaks generated by the Fabry–Pérot effect [81]. The wavelength of each peak maxima in the RIfS spectrum follows the Fabry–Pérot relationship (*i.e.*, 2*n*_eff_*L*_p_ = *m*λ); where *n*_eff_ is the effective refractive index of NAA; *L*_p_ is the pore length; and *m* is the order of the RIfS fringe; the maximum of which is located at the wavelength, λ. These RIfS peaks are the base of label-free optical biosensors capable of monitoring in real time and *in situ* binding events of biomolecules. These can be detected through changes in the effective optical thickness of NAA, which are translated into shifts in the RIfS peak positions [59,79]. These sensing devices can perform highly sensitive qualitative and quantitative detection of a broad range of analytes. In the past few years, RIfS-NAA platforms have been rapidly developed and used in numerous biosensing applications, including the detection of gases, organic molecules and biomolecules [59,82]. For instance, Pan *et al.* [57] demonstrated the capability of RIfS-NAA sensing platforms for the label-free detection of complementary DNA. Alvarez *et al.* [59] reported on a label-free RIfS-NAA immunosensor to selectively monitor binding events between antibodies and antigens. Their results confirmed that only selective antibody-antigen binding events can be detected through a significant change in the effective optical thickness of the NAA film. This demonstrates the capability of RIfS-NAA platforms to design selective and sensitive, label-free immunosensors for a broad variety of applications. Kumeria *et al.* [58] developed an RIfS-NAA microchip biosensor capable of detecting and quantifying circulating tumour cells (CTCs). In this system, NAA platforms were coated with gold and chemically functionalised with biotinylated anti-EpCAM antibodies [58]. This device was able to capture and detect CTCs in a single step. These authors also demonstrated the ability of this system to carry out gas sensing of volatile sulphur compounds in oral malodour and hydrogen sulphide for biomedical applications. In that study, RIfS-NAA platforms were modified with metal and chemical-sensitive layers with affinity towards gas molecules [56].

**Figure 8:**
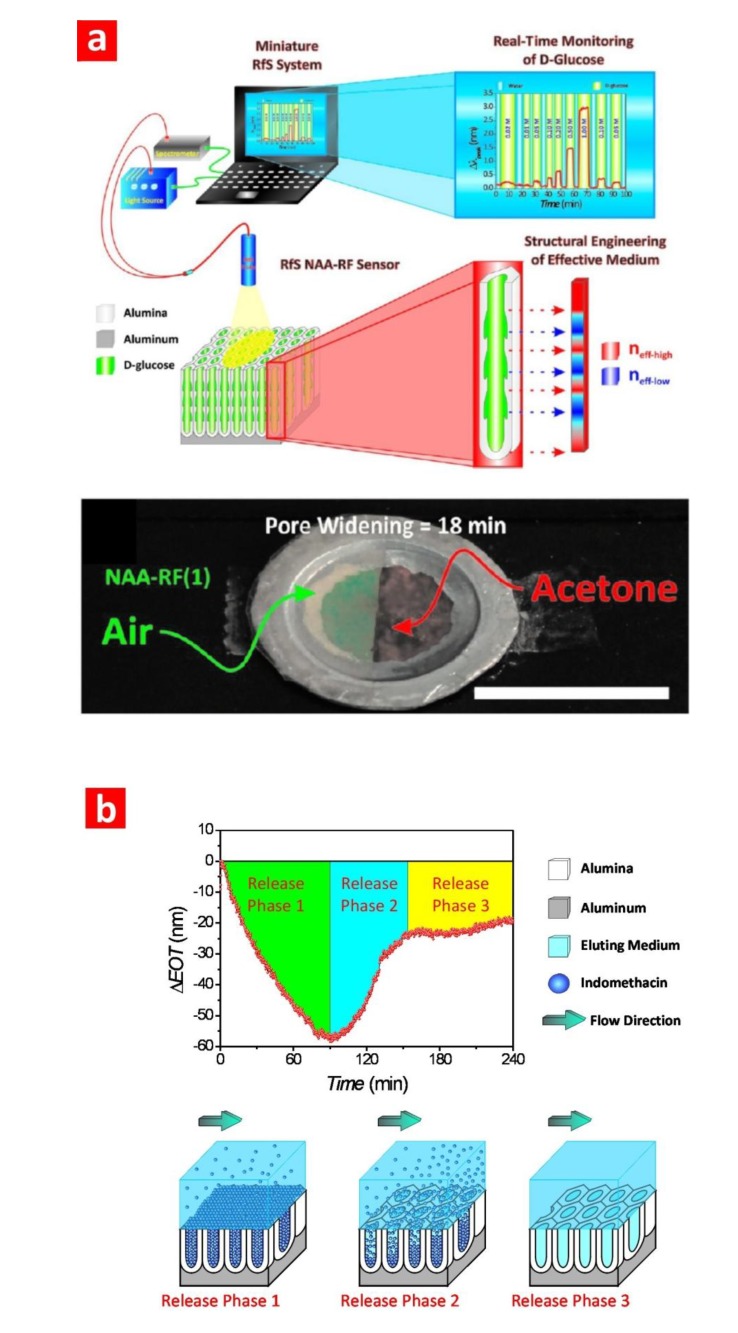
(**a**) Optical system developed by Kumeria *et al.* using RIfS-NAA platforms based on optical rugate filters to detect different levels of glucose (adapted from [2]). (**b**) Characteristic drug release profile of indomethacin from RIfS-NAA platforms under flow conditions (adapted from [62]).

Dronov *et al.* [62] developed an interferometric transducer based on platinum-coated NAA. They demonstrated that the metal coating significantly improves the interferometric fringe patterns, increasing the signal-to-noise ratio. They compared these RIfS-NAA platforms with porous silicon ones, demonstrating that NAA is more sensitive than porous silicon with stable signals. Furthermore, they assessed the sensing capability of this system by detecting two immunoglobulin antibodies, anti-BSA and anti-human IgG. Another interesting study was reported by Santos *et al.* [55], who compared the sensing performance of PL-NAA and RIfS-NAA sensing platforms when detecting different analytes under distinct adsorption conditions. First, NAA platforms were structurally engineered in order to optimise the optical signals obtained by PL and RIfS. Then, the most optimal PL-NAA and RIfS-NAA platforms were quantitatively compared by detecting two different analytes, D-glucose and L-cysteine, under non-specific and specific adsorption conditions, respectively. The obtained results demonstrated that the nature of the analyte molecules and the adsorption conditions in which these molecules are immobilised onto the nanoporous platform play a direct role in the sensing performance. Furthermore, it was verified that PL-NAA platforms show better linearity, higher sensitivity toward analytes and a lower limit of detection than those of RIfS platforms. More recently, Kumeria *et al.* [60] have presented a study on an ultrasensitive sensor based on RIfS-NAA platforms for detecting gold(III) ions, which are relevant in some clinical applications (e.g., gold-based drugs and bioimaging probes). In this system, NAA platforms were functionalised with 3-mercaptopropyl-tirethoxysilane (MPTES) in order to selectively detect gold ions. The change in the effective optical thickness of the NAA platform in RIfS was used as the sensing parameter to characterise these sensors. The linear range, low limit of detection, saturation concentration, selectivity and real-life application of these RIfS-NAA platforms was assessed through a series of experiments. Macias *et al.* [61] demonstrated a funnel-like RIfS-NAA sensing platform featuring a bilayered structure. The resulting RIfS spectrum of these platforms showed a complex series of Fabry–Pérot interference fringes. This biosensing platform was assessed by detecting BSA molecules. Kumeria *et al.* [2] demonstrated the structural engineering and optical optimisation of NAA rugate filters combined with RIfS for the detection of levels of glucose (Figure 8a). This optical biosensing system is based on shifts in the characteristic peak position of the rugate filters produced by small changes in the effective medium (*i.e.*, refractive index). The obtained results were validated by a theoretical model (*i.e.*, the Looyenga–Landau–Lifshitz model), demonstrating that the control over the nanoporous structure makes it possible to optimise optical signals in RIfS for biosensing applications.

Notice that RIfS-NAA platforms not only can be used to monitor binding events between analyte molecules and surface functional groups, but also to study the release of molecules from NAA platforms. Kumeria *et al.* [83] used this approach to characterise the release of a model drug, indomethacin, under dynamic flow conditions through changes in the effective optical thickness of the NAA platform (Figure 8b). The obtained results revealed that this process is controlled by a diffusion mechanism, and the drug release depends on the flow rate; and the faster the flow rate, the higher the drug release from the nanoporous platform. This approach provides more reliable information than conventional *in vitro* drug release techniques performed under static conditions.

## 4. Conclusions

This review has summarised recent progress in the use of NAA platforms to develop optical biosensing systems. We have presented in detail the fundamental aspects of each optical technique used in combination with these nanoporous platforms and provided relevant concepts and examples of devices based on different optical sensing principles. As these studies have demonstrated, the key features of NAA platforms (e.g., cost-competitive and scalable fabrication process, well-defined and versatile nanoporous structure, chemical and physical stability, optical activity, *etc.*) make them an outstanding and promising alternative to widely used porous silicon platforms. NAA is a highly attractive nanomaterial for developing a broad range of optical biosensing systems for clinical, industrial and environmental analyses. NAA can be structurally engineered to provide complex nanostructures with optimised optical properties. Furthermore, this material can be modified with desired functionalities to endow optical biosensors with high selectivity towards target analytes. It is expected that further development and progress in structural and chemical modifications of NAA will enable the development of innovative optical sensing systems based on NAA, featuring up-to-the-minute capabilities. Finally, this review has demonstrated that there is still an excellent opportunity for further advances and developments of optical sensing systems based on NAA, in particular through further miniaturisation and integration into lab-on-chip systems for real-life applications.
